# Recovery of *Bacillus licheniformis* Alkaline Protease from Supernatant of Fermented Wastewater Sludge Using Ultrafiltration and Its Characterization

**DOI:** 10.4061/2011/238549

**Published:** 2011-08-18

**Authors:** Jyothi Bezawada, S. Yan, Rojan P. John, R. D. Tyagi, R. Y. Surampalli

**Affiliations:** ^1^INRS-ETE, Université du Québec, 490, Rue de la Couronne, QC, Canada G1K 9A9; ^2^USEPA, P.O. Box-17-2141, Kansas City, Kansas, KS 66117, USA

## Abstract

Investigation on recovery of alkaline protease from *B. licheniformis* ATCC 21424 fermented wastewater sludge was carried out by centrifugation and ultrafiltration. Optimization of ultrafiltration parameters (transmembrane pressure (TMP) and feed flux) was carried out with 10 kDa membrane. TMP of 90 kPa and feed flux of 714 L/h/m^2^ gave highest recovery (83%) of the enzyme from the centrifuged supernatant. The recovered enzyme had given maximum activity at temperature of 60°C and at pH 10. It was stable between pH 8 to 10 and retained 97% activity at 60°C after 180 min of incubation. Enzyme activity was significantly augmented by metal ions like Ca^2+^ and Mn^2+^. Protease inhibitors like phenylmethyl sulphonyl fluoride (PMSF) and diisopropyl fluorophosphates (DFPs) completely inhibited the enzyme activity. The partially purified protease showed excellent stability and compatibility with various commercial detergents. The detergent (Sunlight) removed the blood stains effectively along with the enzyme as additive.

## 1. Introduction

The membrane separation processes are the most widespread in the field of biotechnology, and they are more easily operated and scaled up in comparison to other bioseparation processes such as chromatography, and electrophoresis. Among the various membrane separation processes, ultrafiltration is one of the processes that functions under pressure gradient which is mostly used for separation and purification of products including enzymes and other proteins [1–3] or to recover microbial products (cells and spores) present in a culture medium [4–6]. Because of the low amount of enzyme present in the cell-free filtrate, the water removal is a primary objective. Ultrafiltration is an effective technique that has been largely used for the recovery of enzymes [7, 8] and, in general, is a preferred alternative to evaporation. This pressure driven separation process is not expensive and also gives encouraging results with little loss of enzyme activity. This process offers both concentration and purification [9]. However, the application of membrane processes in general have some specific problems like fouling or membrane clogging due to the precipitates formed by the final product and/or deposition of solid particles on the membrane. If the solute flow towards the membrane is greater than the solute passing through the membrane, the solute accumulates on the surface of the membrane, this accumulation forms a concentration layer which is known as concentration polarization [10]. Tangential flow filtration is powerful and advantageous alternative over normal flow filtration as tangential flow filtration will significantly reduce the fouling of the membrane. The clogging or fouling can usually be alleviated or overcome by treatment with detergents, proteases, acids or alkalis [11]. In fact, the ultrafiltration process has been effectively in use for the recovery of organic compounds from several synthetic media [12–14]. 

Proteases are commercially important industrial enzymes accounting 60% of the total enzyme sales with two thirds of the proteases produced are from microorganisms [15–17]. Microbial enzymes are replacing chemical catalysts in manufacturing chemicals, food, leather goods, pharmaceuticals, and textiles. Among proteases, alkaline proteases are employed mainly as detergent additives because of their distinctive abilities to assimilate proteinaceous stains such as blood, chocolate, and milk. Currently, alkaline protease-based detergents are preferred over the conventional synthetic detergents, as they have better cleaning properties, higher efficiency at lower washing temperatures, and safer dirt removal conditions [18]. Preferably, proteases used in detergent formulation must have a high activity level and stability over a wide range of pH and temperature. One of the major drawbacks affecting the stability of enzymes recovered from thermophiles at alkaline pH is that enzymes from alkalophiles confer stability over wide pH range but are generally thermolabile. So, there is always a need for proteases with all desirable properties to become accustomed with application conditions, and also, it is necessary to check the stability of the enzyme at elevated temperatures and pH. Applications, such as protease for detergent industries need concentrated and cleaned enzyme to amend with detergent to get good performance during storage and application as well. The enzyme is cleaner when the medium is simple and defined, where, as in case of sludge medium, fermented enzyme contains many other sludge particles and other impurities, so enzyme has to be clarified and concentrated to get higher activity. 

Huge amount of municipal wastewater sludge has been generating in Canada. Due to increase in population and other developments sludge management is becoming a crucial environmental concern due to strict regulations on sludge disposal. So, bioconversion of wastewater sludge into value added products is economic and ecofriendly approach. The use of wastewater sludge for the production of alkaline protease with *Bacillus licheniformis* has been successfully achieved in our laboratory [19, 20]. 

The aim of the present study was to recover and concentrate the alkaline protease activity from culture filtrate of fermented wastewater sludge using ultrafiltration technique. The efficiency of enzyme was examined in the presence of standard commercial detergents and the enzyme was characterized with respect to the effect of various additives such as stabilizers and inhibitors on the stability at higher temperatures and in alkaline pH. 

## 2. Materials and Methods

### 2.1. Bacterial Strain

*Bacillus licheniformis* strain ATCC 21424 was used in this study. An active culture was maintained by inoculating on nutrient agar (composition: 0.3% beef extract, 0.5% peptone, and 2% agar) plates and incubating at 35°C for 48 h. The plates were stored at 4°C for later use.

### 2.2. Sludge Samples and Composition

The wastewater secondary sludge samples collected from municipal wastewater treatment of Communauté Urbaine de Quebec (CUQ, Quebec) were used. The experiments were conducted at a sludge suspended solids concentration of 30 g/L. The sludge was centrifuged in order to obtain higher suspended solids concentration (30 g/L). The sludge characteristics were measured according to standard methods [21] and sludge composition was presented in Table 1. 

### 2.3. Inoculum and Cultural Conditions

A loopful of bacterial growth from a nutrient agar plate was used to inoculate a 500 mL Erlenmeyer flask containing 100 mL of sterilized nutrient broth (composition: 0.3% beef extract and 0.5% peptone) (sterilized at 121°C for 15 min). The flask was incubated in a shaker incubator (New Brunswick) at 35°C with 220 rpm for 12 h. 500 mL Erlenmeyer flasks containing 100 mL of sterilized sludge were then inoculated with 2% (v/v) inoculum from the above flask. The flasks were incubated in the same way for 12 h and these actively growing cells were used as inoculum for fermentor experiments.

### 2.4. Fermentation

A fermenter (Biogénie Inc., Quebec) of 15 L capacity equipped with accessories and automatic control systems for dissolved oxygen, pH, antifoam, impeller speed, aeration rate and temperature and with working volume of 10 L sludge supplemented with 1.5% (w/v) soybean meal and 1.5% (w/v) lactose (sterilized at 121°C for 30 min) was used for production of extracellular alkaline protease. The medium was inoculated with 4.5% (v/v) inoculum. Temperature and pH of the fermentation medium were controlled at 35°C and 7.5, respectively. Dissolved oxygen concentration was maintained above 20% (1.56 mg O_2_/L) saturation (critical oxygen concentration) by agitating the medium initially at a speed of 200 rpm and finally increased up to 500 rpm and air flow rate was controlled automatically using a computer controlled system. The fermented broth was collected aseptically in HDPE bottles (VWR Canlab, Canada) after 42 h of fermentation and sealed with paraffin and preserved at 4°C for further use.

### 2.5. Techniques for Recovery of Alkaline Protease from the Fermented Broths

#### 2.5.1. Centrifugation

Fermented broth was centrifuged at 9000x g for 30 min according to the procedure of Brar et al. [22]. The supernatant after the centrifugation of the fermented broths were collected and stored at 4°C.

#### 2.5.2. Ultrafiltration

**Operating Principle and Washing of the Filter**

The equipment used for ultrafiltration was of tangential flow filtration type (PREP/SCALE-TFF, Cartridges Millipore) with recirculation. The fluid was tangentially pumped along the surface of the membrane. Pressure was applied to force a portion of the fluid through the membrane to the permeate side. The supernatant from centrifuge was fed into the ultrafiltration equipment by a pump (Casy Load, Master Flex, Millipore). The supernatant was brought to room temperature (25°C) in order to conduct ultrafiltration study. The process consisted of feeding aseptically a volume (1 L) of the supernatant from the centrifugation step referred to as “feed” through the membrane in order to concentrate the active components to a concentrated volume referred to as “retentate” which was 18% of the volume of the supernatant [28]. The flow of the supernatant was obtained by means of a pump whose flow varied between 45.5 L/h and 250 L/h, which gave a flow of feed through the membrane ranging between 455 L/h m^2^ and 2500 L/h/m^2^. As for the flow of permeate, it generally depended on the TMP and the resistance of the membrane. After ultrafiltration, the permeate and retentate were collected in flasks. Sampling of the supernatant, retentate, and permeate was carried out for measurements of physical and biological parameters (total solids, suspended solids, soluble protein, and protease activity).

After each ultrafiltration operation, liquid in the membrane was completely drained. Taking into account the type of medium used in this study (biological environment), it was recommended to use an alkaline solution (0.1 N NaOH). The alkaline solution was passed through the membrane until the membrane was clean. Later, the membrane was removed and inverted to facilitate complete washing. Resistance of the membrane can be determined by “normalized permeability weight” (NWP) *as* performance of the membrane depends on NWP. The NWP can be calculated by (1). In fact, during the use of the membrane, the value of NWP decreased, and when the value lies between 10% and 20% of its initial value (that of the new membrane, NWP of new membrane should be considered as 100%), the membrane should then be changed [6]. Prior to ultrafiltration runs, we have determined the NWP to check the resistance of the membrane.

(1) $$\begin{array}{cc} \text{NWP} & =\frac{\text{water filtrate flux}}{\text{TMP (Transmembrane pressure (15 Psi))}\times {\text{TCF}}_{{\text{20}}^{{}^{\circ}}\text{C}}\left(\text{temperature correction factor}\right)}. \end{array}$$

**Membrane Size**

Membranes with molecular weight cut-off (MWCO) of 10 kDa and 100 kDa were used in the present study (Millipore, prep/scale spiral wound TFF-1). The membrane was made up of regenerated cellulose and was of the type spiral wound TFF-1 module PLCC with a surface area of 0.1 m^2^. Supernatant was passed first through 100 kDa membrane to eliminate all other sludge impurities and final permeate was collected as enzyme source to carry out optimization studies using membrane of 10 kDa. Concentrated enzyme was used for characterization purpose. Characteristics of the membranes were presented in Table 2.

**Optimization of Parameters of Ultrafiltration**

Transmembrane pressure and flux of the feed are important parameters to be controlled in ultrafiltration. For the optimization, the experiment was carried out for various values of TMP (70–110 kPa) and feed fluxes (455–2500 L/h/m^2^). In a typical ultrafiltration process, lower permeate flow results in higher solute concentration in the retentate. Samples were withdrawn to determine the suspended solids, total solids, soluble protein, and protease activity in the retentate and permeate.

### 2.6. Analysis of Parameters

#### 2.6.1. Physical Parameters

The total solids of the samples (supernatant, retentate, and permeate) were measured by drying 30 mL volume at 105°C [21]. The suspended solids were determined by filtration through the 0.45 *μ*m membrane (Glass Microfiber Filter 934-AH of 42.5 mm, Whatman) followed by drying at 105°C. The determination of the soluble protein concentration of the samples (supernatant, retentate and permeate) was carried out using Braford method [23] with a spectroscopic measurement of the absorbance at 595 nm. All the experiments were conducted in triplicates and the mean value was presented.

#### 2.6.2. Alkaline Protease Activity

The modified method of Kunitz [24] was used to determine Protease activity. Fermented samples were centrifuged and the supernatant was collected as the crude enzyme source. The supernatant thus obtained was properly diluted with borate buffer (pH 8.2). Protease activity was assayed by incubating 1 mL of properly diluted enzyme solution with 5 mL of casein (1.2% w/v, Sigma-Aldrich Canada Inc) for 10 min at 37°C in a constant temperature water bath. The reaction was terminated by adding 5 mL of 10% (w/v) trichloroacetic acid. This mixture was incubated for 30 min in order to precipitate the total nonhydrolyzed casein. Samples and blanks were filtered using Whatman filter paper (934-AH) after the incubation period. The absorbance of the filtrate was measured at 275 nm. The validation of the results was established by treating a standard enzyme solution under identical experimental settings where activity was known. One international protease activity unit (IU) was defined as the amount of enzyme preparation required to liberate 1 *μ*mol (181 *μ*g) of tyrosine from casein per minute at pH 8.2 and 37°C. All experiments were conducted in triplicate and the mean value was presented.

### 2.7. Characterization of Partially Purified Enzyme

#### 2.7.1. Effect of pH on Enzyme Activity and Stability of Protease

The activity of protease was measured at different pH values in the absence and presence of 10 mm CaCl_2_. The pH was adjusted using different buffers; acetate buffer (pH 5), phosphate buffer (pH 6-7), borate buffer (pH 8-9), bicarbonate buffer (pH 10), and Robinson and stokes buffer (pH 11-12). Reaction mixtures were incubated at 37°C and the activity of the enzyme was measured.

Stability of the enzyme was determined by incubating the reaction mixtures at various pH values using different relevant buffers (pH 5–12) for 2 h at 37°C. The residual activity after incubation was determined under standard assay conditions. Residual activities are obtained at respective pH values assuming the activity of enzyme before the incubation is 100%.

#### 2.7.2. Effect of Temperature on Activity and Stability of Protease

Optimum temperature was determined by activity assay on casein at pH 10 from 30°C–90°C in the absence and presence of 10 mm CaCl_2_ and relative protease activities were assayed at standard assay conditions using casein as substrate.

The thermostability of enzyme was measured by incubating the enzyme preparation at different temperatures ranging from 30°C–90°C for 180 min in the absence and presence of 10 mm CaCl_2_. The residual activity after incubation was determined under the standard assay conditions. Residual activities are obtained at respective temperatures assuming the activity of the enzyme before the incubation is 100%.

#### 2.7.3. Effect of Enzyme Inhibitor and Chelator on Protease Activity

The effect of various protease inhibitors (5 mm) such as serine inhibitors (phenylmethyl sulphonyl fluoride [PMSF] and diisopropyl fluorophosphate [DFP]), cysteine-inhibitors (p-chloromercuric benzoate [*p*CMB] and *β*-mercaptoethanol [*β*-ME], iodoacetate), dithiothreitol, and a chelator of divalent cations (ethylene diamine tetra acetic acid [EDTA]) on enzyme activity were investigated by preincubation with the enzyme solution for 30 min at 60°C. The relative protease activity was measured under assay conditions.

#### 2.7.4. Effect of Various Metal Ions on Enzyme Activity

To study the effect of various metal ions (Ca^2+^, K^+^, Fe^2+^, Zn^2+^, Hg^2+^, Mg^2+^, Mn^2+^, Cu^2+^, Co^2+^, Na^+^) on enzyme activity, metal salt solutions were prepared in a concentration of 10 mm and 1 mL of metal solution was mixed with 5 mL of enzyme and was incubated for 2 h. Enzyme activities were measured at standard assay conditions. The activity is expressed in terms of relative activity assuming that the activity of the enzyme in the absence of metal salts just before the initiation of the treatment is 100%.

#### 2.7.5. Hydrolysis of Protein Substrates

The effect of various protein substrates such as casein, BSA, egg albumin, and gelatin were determined under assay conditions by mixing 1 mL of the enzyme and 5 mL of assay buffer containing the protein substrates (1.2% w/v). After incubation at 60°C for 10 min, the reaction was stopped by adding 10% TCA (w/v). The undigested protein was removed by filtration (whatman filter paper, 934-AH) and the absorbance of the filtrate was measured at 275 nm. The protease activity towards casein was taken as a control.

#### 2.7.6. Effect of Detergents on Stability of Protease Activity

Protease enzyme stability with commercial detergents was studied in the presence of 10 mm CaCl_2_. The detergents used were Merit Selection (Metrobrands, Montreal, Quebec), La Parisienne (Lavo Inc., Montreal, Quebec), Arctic Power (Phoenix brands Canada), Bio-vert (Savons Prolav Inc., Canada) and Sunlight (the Sun products of Canada corporation, Ontario). The detergent solutions (0.7% w/v) were prepared in distilled water and incubated with the partially purified enzyme (2 mL recovered enzyme and 1 mL detergent of 0.7%) up to 3 h at 60°C. At every 30 min interval, the protease activity was estimated under standard assay conditions. The control was maintained without any detergent and enzyme activity was taken as 100%.

### 2.8. Application of Alkaline Protease in Removing Blood Stains

Application of protease enzyme (2 mL recovered enzyme) as detergent additive in removing blood stains was studied on white square cotton cloth pieces measuring 4 × 4 cm prestained with goat blood according to the method of Adinarayana [17]. The stained cloths were air dried for 1 h to fix the stain. Each of these stained cloth pieces were taken in separate flasks and the following setups were prepared.

1. Flask with distilled water (100 mL) + stained cloth.
2. Flask with distilled water (100 mL) + stained cloth + 1 mL of respective detergent solution at 7 mg/mL.
3. Flask with distilled water (100 mL) + stained cloth + 1 mL of respective detergent solution (7 mg/mL) + 2 mL of enzyme solution (114 IU/mL).

The above flasks were incubated at 60°C for 15 min. After incubation, cloth pieces were taken out, rinsed with water, and dried. Visual examination of various pieces exhibited the effect of enzyme in removal of stains. Untreated cloth pieces stained with blood were taken as control. 

## 3. Results and Discussion

### 3.1. Optimization of Parameters of UF

#### 3.1.1. Transmembrane Pressure (TMP)

The TMP was given by [6]

(2) $$\text{TMP}=\left[\frac{P_{\text{feed}}+\;P_{\text{net}}}{2}\right]-P_{\text{perm}}.$$

**Figure 1:**
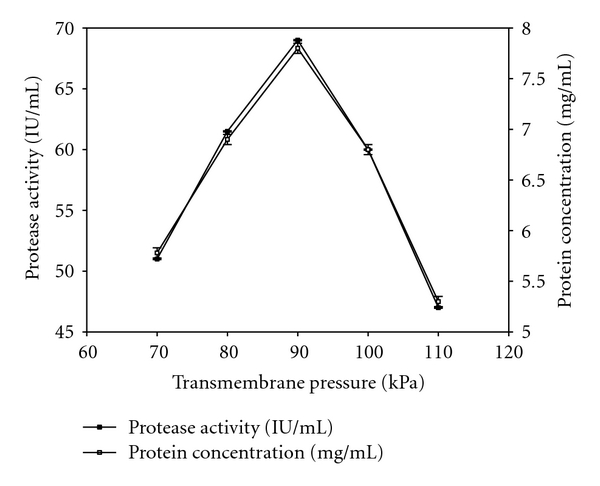
Protease activity and protein concentration in the retentate at different transmembrane pressures.

**Figure 2:**
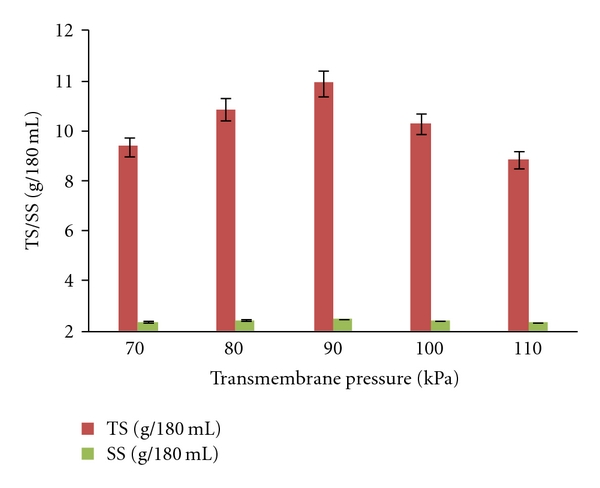
Total solids (TS) and suspended solids (SS) concentration in the retentate at different transmembrane pressures.

TMP is function of the pressure of the retentate and that of feed which is adjusted by pressure gauge in order to get different values of TMP. Different TMP values were tested range from 70 kPa to 110 kPa to get the optimum TMP value. Profiles of protease activity, soluble protein, total solids, and suspended solids in the retentate were presented in Figures 1 and 2, respectively. All of these values (protease activity, soluble protein, total solids, and suspended solids) were not detectable in permeate as the size of alkaline proteases were in range between 20–30 kDa [11] which is larger than MWCO of 10 kDa membrane. Adjalle et al. [6] reported negligible values of viable spores, total cells and turbidity in the different ultrafiltrated permeates of different fermented broths as sizes of spores and cells of *B. thuriegensis* (25 kDa and 30 kDa) during recovery of biopesticide are greater than MWCO of 5 kDa membrane. Higher protease activity (69 IU/mL), soluble protein (7.8 mg/mL), total solids (12 g/L), and suspended solids (2.7 g/L) were obtained with 90 kPa among all other TMP values used. All tested components were concentrated to 4.5 times in the retentate than their initial values (supernatant) (Table 3). The fact that the protease activity was not detectable in all permeates. So, 83% of protease activity was recovered in retentate with TMP of 90 kPa. Therefore, it is apparent that some of the protease was lost as a deposit on the membrane and/or in the tubes. TMP values higher or lower than 90 kPa were resulted in lower values of protease activity, soluble protein, total solids, and suspended solids. This may be because when TMP is lower than optimum value, feed pressure may not be sufficient to pass the solution through the membrane effectively and loss of components might have occurred in tubes or on the membrane surface. When TMP was high, this high pressure had cause foaming in ultrafiltration membrane which was retained in tubes and eventually components loss on membrane and in tubes might have occurred in the form of foam as they cannot be retained either in the permeate or in the retentate. Flow of the solute across the membranes certainly leads to clogging of some of the pores, creating additional surfaces for adsorption and caking. As gentle conditions are required for the recovery of the intact proteins, it would be difficult to remove/recover these proteins. Various mechanisms could generate these losses through the ultrafiltration membrane, as some of the sample components are close to the MWCO of the membranes. Primary cause of the loss of the enzyme protein through the membrane is the pore size distribution, while shear forces could also contribute by producing smaller fragments [25]. According to ultrafiltration principle, minimum flow of permeate will result in minimum loss or no loss of solute (protease in the present context) in the permeate and will give high concentration in retentate. Minimal flow of the permeate can be obtained with an optimal value of the TMP [6]. Adjalle et al. [26] recorded TMPs of 90 kPa and 100 kPa were optimum values for entomotoxicity recovery from starch industry wastewater and thermal hydrolyzed sludge medium, respectively. Other researcher reported that 20 kPa was best for the separation of serine alkaline protease from neutral protease and amylase and 100 kPa for the separation of serine alkaline protease from the organic and amino acids [3]. The optimum TMP is different for different cases and may be due to the rheological characteristics and other components present in the feed samples.

#### 3.1.2. Feed Flux

**Figure 3:**
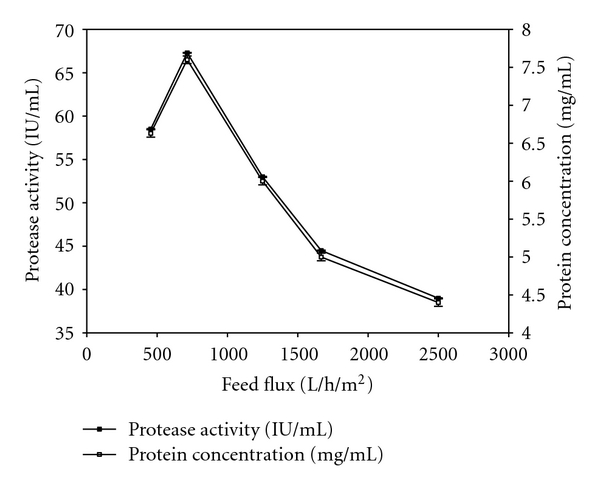
Protease activity and protein concentration in the retentate at different feed flux rates.

**Figure 4:**
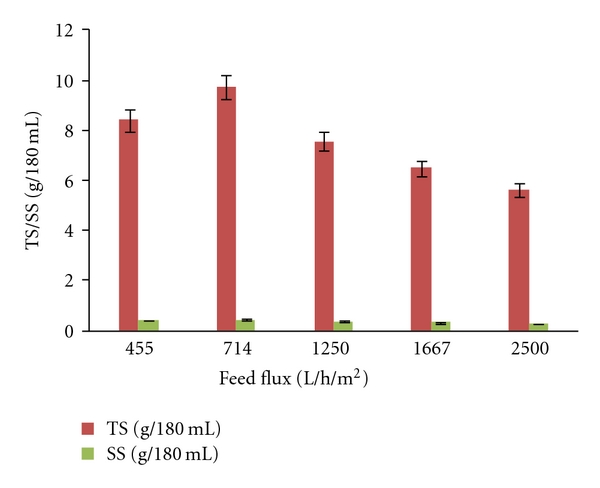
Total solids (TS) and suspended solids (SS) concentration in the retentate at different feed flux rates.

Profiles of protease activity, soluble protein, total solids, and suspended solids versus different feed fluxes at TMP of 90 kPa were presented in Figures 3 and 4. Different feed flux values between 455 L/h/m^2^ and 2500 L/h/m^2^ were used by keeping the circulation of fluid at flow rate between 45.5 L/h (lowest speed) and 250 L/h (highest speed). During feed flux optimization, for each feed flux value, the retentate pressure was adjusted in order to maintain same TMP of 90 kPa. The feed flux of 714 L/h/m^2^ gave highest values of protease activity, soluble protein, total solids, and suspended solids in the retentate among all feed fluxes used. All of these values were not detectable in all permeates as explained earlier. Protease activity, soluble protein, total solids, and suspended solids were concentrated to 4.5 times than their initial values (supernatant) (Table 3). 81% of the protease (in terms of activity) was recovered in the retentate at feed flux of 714 L/h/m^2^. No protease was detected in the permeate as most of the protease was recovered in the retentate and some of the protease might get lost as deposit on the membrane. Li et al. [27] reported that purities of proteases were increased more than ten times at flow rate of 360 L/h while separation of proteases from yellow fin tuna spleen by ultrafiltration, and Adjalle et al. [26] reported 550 L/h/m^2^ and 720 L/h/m^2^ as optimal values for entomotoxic components (crystal protein, protease etc.) from starch industry wastewater and thermal hydrolyzed sludge medium, respectively. Protease activity, protein, total solids, and suspended solids concentrations were decreased with higher feed flux values (1250, 1667 and 2500 L/h/m^2^). This decrease with high feed flux values due to the fact that high flux can degrade product quality due to the generation of turbulence effect [28]. Moreover, higher turbulence can cause severe foam in the retentate stream which will create a vacuum and further decrease the permeate flux below the optimum value; hence, administer the overall performance of the ultrafiltration system [6]. The vibration in the filtration unit due to the higher feed flux can cause foaming due to proteins (enzymes and other soluble proteins) present in the medium [29].

### 3.2. Characterization of Partially Purified Enzyme

#### 3.2.1. Effect of pH on Enzyme Activity and Stability of Protease

**Figure 5:**
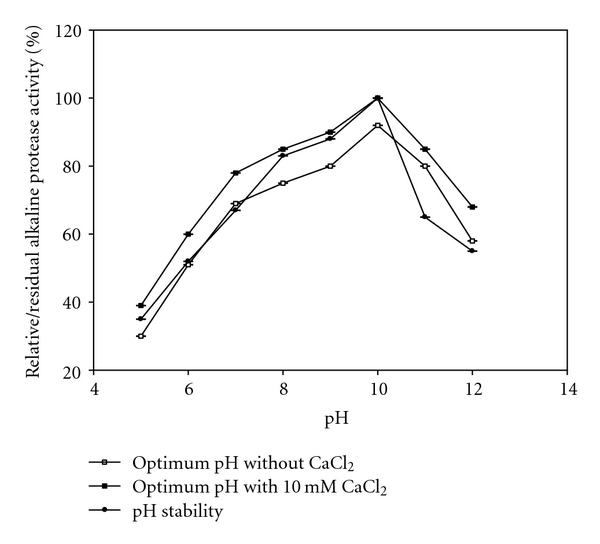
Effect of pH on the activity and stability of alkaline protease in the absence and presence of 10 mM CaCl_2 _.

pH is a determining factor in the expression of an enzyme activity as it alters the ionization state of the amino acid or ionization of substrate. The ionization state of enzymes is undoubtedly one of the most crucial parameters that control substrate binding, catalytic enzyme action, and three-dimensional structure of enzyme molecule. Effect of pH on enzyme activity (permeate of 100 kDa) and stability in the presence and absence of 10 mm CaCl_2_ are presented in Figure 5. The partially purified protease was found to be typical alkaline protease displaying its maximum activity at alkaline pH 10 as optimum, and it decreased sharply with increase in pH. The active site of the enzyme is mainly composed of ionic groups that must be in proper ionic form to maintain the conformation of the active site of enzyme for substrate binding or reaction catalysis as conformation is sensitive to changes in the environment (like pH change) [30]. The optimum pH obtained for this enzyme was higher than other reports showing pH optimum of 8 for protease from *Haloferax lucentensis* VKMM 007 [31] and pH optimum of 9 for protease from *B. stearothermophilus* [15]. However, the findings of optimum pH for this enzyme was in accordance with other findings who also reported pH of 10-10.5 as optimum for proteases from *B. subtilis* PE-11, *B. cereus* and *Vibrio metschnikovi* [17, 32, 33]. Protease stability of concentrated protease in presence and absence of 10 mm CaCl_2_ is shown in Figure 5. When protease was preincubated with various buffers over broad pH values (pH 5–12) for 2 h and then measured for residual activity, the protease showed highest stability over a broad range of pH 8 to 10. The enzyme stability declined to nearly 55% when pH values were higher than 10. The most important feature of the present protease is alkaline enzyme, as it was stable in the alkaline pH up to 11, and it can be used as additive in detergent industry. Usually, commercially important proteases from microorganisms have highest activity in the alkaline pH range of 8–12 [40, 45].

#### 3.2.2. Effect of Temperature on Activity and Stability of Protease

**Figure 6:**
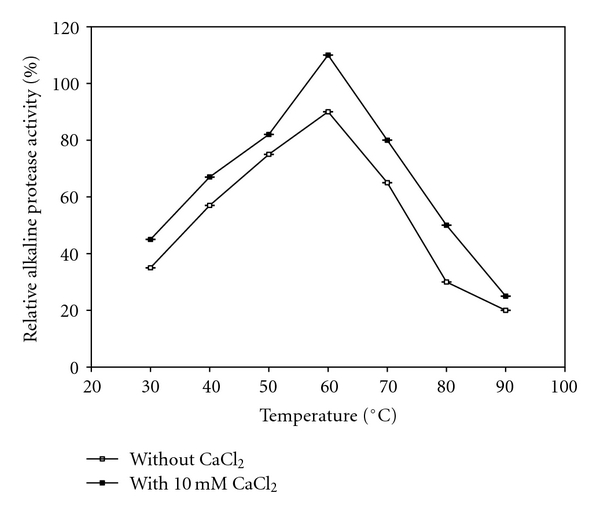
Effect of temperature profiles on activity of enzyme in the absence and presence of 10 mM CaCl_2 _.

Temperature profiles on enzyme activity in the presence and absence of 10 mm CaCl_2_ are shown in Figure 6. In the present study, temperatures ranging from 30°C–90°C were studied in the absence and presence of 10 mm CaCl_2_. Optimum temperature for this enzyme was found to be 60°C. The enzyme activity was declined gradually when temperatures were higher than 60°C. Similar results were reported by other researchers where optimum temperature of 60°C was recorded for proteases from *Haloferax lucentensis* VKMM 007 and *B. mojavensis* [31, 34]. In contrary to these results, an optimum temperature of 75°C was reported for protease of *B. laterosporus*-AK1 [35]. 

**Figure 7:**
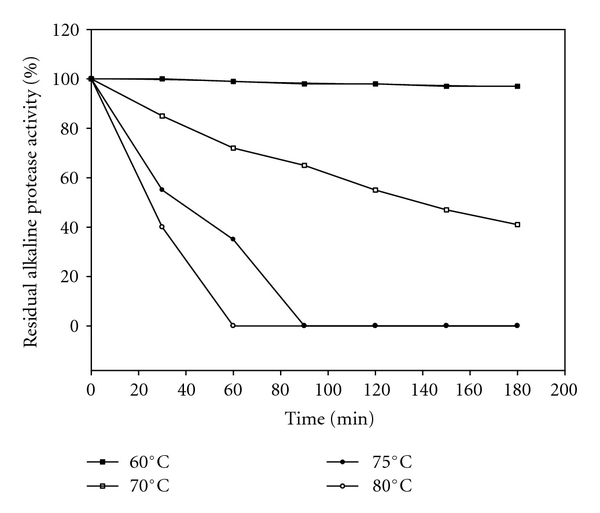
Effect of temperature on stability of alkaline protease enzyme (ultrafiltrated samples) in the presence of 10 mM CaCl_2_.

Temperature profile on enzyme stability was presented in Figure 7. Thermal stability of the enzyme was studied at different temperatures of 60°C, 70°C, 75°C, and 80°C for different time periods (30 to 180 min) in the presence of 10 mm CaCl_2_. The enzyme was 97% stable at 60°C even after 180 min of incubation and at 70°C up to 41% stable after 180 min incubation. But, the enzyme was completely unstable at 75°C after 90 min of incubation and at 80°C after 60 min of incubation due to thermal inactivation. The main course of action found to be involved in the thermal denaturation of enzyme was due to the dissociation of ionic groups from the holoenzyme and modification or degradation of prosthetic group [30]. Beg and Gupta [34] reported 86% of stability at 60°C for protease from *B. mojavensis* and Shanmughapriya et al. [36] reported enzyme stability only up to 40°C for protease from marine *Roseobacter* sp. MMD040. The present enzyme from *B. licheniformis* ATCC 21424 is thermostable as thermostable enzymes are stable and active at temperatures which are even higher than the optimum temperatures for the growth of the microorganisms [37]. So, this enzyme can be very well applied as additive in detergent industries, as it can withstand harsh washing conditions of operation because of its stability at high temperatures. 

#### 3.2.3. Effect of Enzyme Inhibitor and Chelator on Protease Activity

Inhibition studies primarily give an insight of the nature of an enzyme, its cofactor requirements and the nature of the active center [38]. The effect of different inhibitors and chelator on enzyme activity was investigated and results were presented in Table 4. Among all the inhibitors tested (5 mm concentration), PMSF inhibited the protease activity completely and DFP was able to inhibit protease activity up to 90%. In this context, PMSF sulphonates the vital serine residue in the active site of the protease and has been reported to inactivate the enzyme activity completely [39]. Hence, this protease can be classified as serine protease. Our results are in accordance with other studies, where protease was completely inhibited by PMSF [17, 31]. EDTA and Diothiotheritol did not inhibit enzyme activity at all, but *p*CMB and *β*-ME inhibited the enzyme activity slightly. 

#### 3.2.4. Effect of Various Metal Ions on Enzyme Activity

Effect of various metal ions with 5 mm concentration on the enzyme activity was tested and results were presented in Table 5. Some metal ions (Ca^2+^ and Mn^2+^) had enhancing effect on the enzyme activity, while other ions (Mg^2+^, Zn^2+^, Cu^2+^, Co^2+^, and Na^+^) had no effect or slight inhibitory effect on the enzyme activity. Some of the metal ions such as Ca^2+^, Mn^2+^, and Mg^2+^ increased and stabilized protease enzyme activity; this may be due to the activation by the metal ions. Results show that these metal ions protected the enzyme activity against thermal inactivation and played a vital role in maintaining active conformation of the enzyme at higher temperatures [40]. Metal ions Hg^2+^ and K^+^ had showed maximum inhibition on the enzyme activity, while Fe^2+^ inhibited the enzyme activity up to 34%. The inhibitory effect of heavy metal ions is well reported in the previous reports and it is well known that ions of mercury react with protein thiol groups and also with histidine and tryptophan residues. Moreover, the disulfide bonds were found to be hydrolytically degraded by the action of mercury [40]. 

#### 3.2.5. Hydrolysis of Protein Substrates

Effect of some native proteins as substrates on enzyme was studied and results were presented in Table 6. Among all substrates, protease showed higher hydrolytic activity (100%) against casein and the enzyme showed moderate hydrolysis of both BSA (54%) and egg albumin (35%). Previous researchers also reported that alkaline proteases showed higher activity with casein [40, 41]. But protease did not show any hydrolysis with gelatin. This may be due to the enzymatic cleavage of peptide bonds of gelatin is difficult because of its rigid structure and the restricted enzyme substrate interaction on the surface of gelatin [42]. Different protein substrates contain different amino acid contents and the assay procedure used in this study by detection of tryptophan and tyrosine. So, lesser hydrolysis of other substrates compared to casein may be due to less quantity of detectable amino acid in hydrolyzed product. For example, gelatin has no tryptophan residue. 

#### 3.2.6. Effect of Detergents on Stability of Protease Activity

Protease used for detergent additive is expected to be stable in the presence of various commercial detergents. The protease from *B. licheniformis* ATCC 21424 was tested for its stability in the presence of different commercial detergents. Excellent stability and compatibility was observed with protease in presence of wide range of commercial detergents (Sunlight, Arctic Power, la Parisienne, Merit Selection and Bio-vert) at 60°C in the presence of CaCl_2_ as stabilizer. The protease showed good stability and compatibility in the presence of Sunlight followed by Arctic Power (Table 7). Significant activity (nearly 50%) was retained by the enzyme with most of the detergents tested even after 3 h incubation at 60°C. Protease from *Streptomyces fungicidicus* MML1614 showed compatibility with various commercial detergents and significant activity was retained only up to 90 min of incubation [15] and protease from *Conidiobolus coronatus *showed compatibility with different commercial detergents at 50°C with 25 mm CaCl_2_ concentration, only 16% activity in Revel, 11.4% activity in Aerial, and 6.6% activity in Wheel after incubation [43].* B. licheniformis* RP1 protease was stable with detergents at 40°C–50°C only up to 60 min [44]. In comparison with these results, our protease from *B. licheniformis *ATCC 21424 was appreciably more stable in the presence of commercial detergents.

### 3.3. Washing Test With *B. licheniformis* Protease in Removing Blood Strains

**Figure 8:**
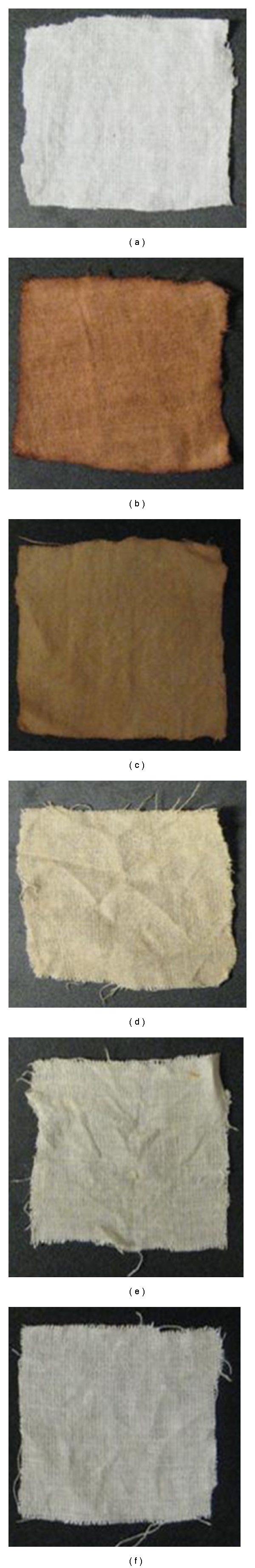
Washing test of alkaline protease from *B. licheniformis* ATCC 21424 in presence of detergent (Sunlight). (a) positive control, (b) negative control, (c) with distilled water, (d) with Enzyme alone, (e) with detergent alone, and (f) with enzyme + detergent.

Protease produced by *B. licheniformis* ATCC 21424 was considerably stable in a wide range of pH and temperature and even showed good compatibility with commercial detergents. So, it was tested as an additive with detergent, to check washing performance of the detergent with this additive protease. When the protease preparation was combined with commercial detergent (Sunlight), washing performance of the detergent was enhanced in removing the blood stain from white cotton cloth (Figure 8). Similarly, proteases of *B. subtilis* [17] and *Streptomyces fungicidicus* [15] efficiently removed blood stains from white cotton cloth pieces when combined with commercial detergents. Even though alkaline proteases from *Bacillus* spp. are stable at high temperature and alkaline pH, most of them are incompatible with detergent matrices [45, 46]. Concentrated enzyme from supernatant of sludge fermented by *B. licheniformis* ATCC 21424 showed higher tolerance with detergent and efficiently removed the blood stain. 

## 4. Conclusion

In this study, enzyme was concentrated and characterized to apply as additive in detergents as detergent industry needs concentrated enzyme in order to have higher efficiency. The recovery of alkaline protease using ultrafiltration process with an optimum transmembrane pressure of 90 kPa and feed flux of 714 L/h/m^2^ showed a recovery of 83% of the protease activity. The protease from *B. licheniformis* ATCC 21424 is thermostable and alkali-tolerant serine alkaline protease, as it is stable at alkaline pH and high temperature. Recovered alkaline thermostable protease by ultrafiltration can be exploited in detergent industry as an additive, because it showed excellent stability at wide range of temperature and compatibility with commercial detergents. More importantly, the supplementation of the enzyme preparation to detergent could remarkably remove the blood stains of white cotton cloth.

## Figures and Tables

**Table 1:** Physical and chemical characteristics of secondary sludge.

Characteristics	Concentration
Physical characteristics:	
Total solids (g/L)	29 ± 1.2
Volatile solids (g/L)	18 ± 0.6
Suspended solids (g/L)	22 ± 0.9
Volatile suspended solids (g/L)	17 ± 0.7
pH	5.7 ± 0.3

Chemical characteristics:	
Total carbon (%, dry total solids)	38.12 ± 1.5
Total nitrogen (%, dry total solids)	5.5 ± 0.3
Ammonical nitrogen (mg N/kg)	680 ± 22.1
Total phosphorus (mg P/kg)	12 422 ± 42
Orthophosphates (mg P/kg)	7 780 ± 24

Metals (in mg/kg) (dry basis):	
Al^3**+**^	13 305 ± 41
Ca^2+^	16 160 ± 45
Cd^2+^	3.5 ± 0.16
Cr^3+^	66.5 ± 2
Cu^2+^	270 ± 9
Fe^2+^	10 365 ± 30
Mg^2+^	1874 ± 62
Mn^2+^	198 ± 6.9
K**^+^**	1720 ± 53
Pb^2+^	61 ± 2
Zn^2+^	477 ± 15

**Table 2:** Characteristics of the membrane.

Description of membrane	Characteristics of membrane
Type	Prep/scale spiral wound TFF-1
Filter type	Ultrafiltration
Length, cm (in.)	23.4 (9.2)
Diameter, cm (in.)	5.8 (2.3)
Minimum working volume (mL)	150
pH range	2.0–13.0
Configuration	Spiral wound cartridge
Filtration area (m^2^)	0.23
Operation temperature (°C)	4–50
Filter material	Regenerated cellulose
Maximum Intel pressure, bar (psi)	0–5.5 (0–80)
Recirculation rate (L/min)	1.0–6.0
Molecular weight cut off (MWCO), kDa	10

**Table 3:** Maximum values of protease activity, soluble protein, total solids, and suspended solids at optimum TMP and feed flux.

Parameters	Supernatant	Retentate	
	Before UF	TMP (90 kPa)	Feed flux (714 L/h/m^2^)
Protease (IU/mL)	15 ± 0.72	69 ± 3.4	67.3 ± 3.3
Soluble protein (mg/mL)	1.7 ± 0.08	7.8 ± 0.39	7.6 ± 0.37
Total solids (g/L)	12 ± 0.6	55.2 ± 2.75 (9.9 g/180 mL	54 ± 2.7 (9.72 g/180 mL)
Suspended solids (g/L)	0.6 ± 0.03	2.7 ± 0.13 (0.48 g/180 mL)	2.52 ± 0.12 (0.45 g/180 mL

**Table 4:** Effect of enzyme inhibitor and chelator on protease activity.

Inhibitor/chelator (5 mM)	Relative enzyme activity (%)
Control	100
*β*-Mercaptoethanol	78 ± 3.8
*p*-chloromercuric benzoate (*p*-CMB)	91 ± 4.3
Phenylmethyl sulphonyl fluoride (PMSF)	0
Diisopropyl fluorophosphate (DFP)	10 ± 0.5
Iodoacetate	96 ± 4.4
Ethelene diamine tetra acetic acid (EDTA)	110 ± 5.6
Dithiotheritol	100 ± 4.8

**Table 5:** Effect of metal ions on alkaline protease activity.

Metal ions (5 mm)	Residual alkaline protease activity (%)
Control	100
Ca^2+^	125 ± 6.1
K^+^	6 ± 0.26
Fe^2+^	66 ± 3.1
Zn^2+^	98 ± 4.7
Hg^2+^	23 ± 1.1
Mg^2+^	99 ± 4.8
Mn^2+^	114 ± 5.5
Cu^2+^	95 ± 4.6
Co^2 +^	91 ± 4.4
Na^+^	96 ± 4.7

**Table 6:** Activity of alkaline protease against different natural proteins.

Protein (2 mg/mL)	Relative enzyme activity (%)
Casein	100
Gelatin	3 ± 0.14
BSA	54 ± 2.6
Egg albumin	35 ± 1.7

**Table 7:** Compatibility of alkaline protease activity from *B. licheniformis* with commercial detergents.

Relative enzyme activity (%)						
Time (h)	Control	Sunlight	La Parisienne	Merit selection	Arctic power	Bio-vert
0.0	100	100	100	100	100	100
0.5	97	95	90	89	91	88
1.0	93	91	82	80	83	81
1.5	90	88	71	69	72	70
2.0	84	81	65	61	64	64
2.5	80	75	57	54	60	59
3.0	74	62	52	48	55	50
